# Sputum Microscopy With Fluorescein Diacetate Predicts Tuberculosis Infectiousness

**DOI:** 10.1093/infdis/jix229

**Published:** 2017-05-16

**Authors:** Sumona Datta, Jonathan M Sherman, Marco A Tovar, Marjory A Bravard, Teresa Valencia, Rosario Montoya, Willi Quino, Nikki D’Arcy, Eric S Ramos, Robert H Gilman, Carlton A Evans

**Affiliations:** 1 Innovation for Health and Development (IFHAD), Laboratory of Research and Development, Universidad Peruana Cayetano Heredia, Lima, Peru;; 2 Infectious Diseases and Immunity and Wellcome Trust Centre for Global Health Research, Imperial College London, United Kingdom;; 3 Innovacion por la Salud y el Desarollo (IPSYD), Asociación Benéfica Prisma, Lima, Peru; and; 4 Department of International Health, Johns Hopkins Bloomberg School of Public Health, Baltimore, Maryland

**Keywords:** fluorescein diacetate, infectiousness, microscopy, tuberculosis, TB

## Abstract

**Background:**

Sputum from patients with tuberculosis contains subpopulations of metabolically active and inactive *Mycobacterium tuberculosis* with unknown implications for infectiousness.

**Methods:**

We assessed sputum microscopy with fluorescein diacetate (FDA, evaluating *M. tuberculosis* metabolic activity) for predicting infectiousness. *Mycobacterium tuberculosis* was quantified in pretreatment sputum of patients with pulmonary tuberculosis using FDA microscopy, culture, and acid-fast microscopy. These 35 patients’ 209 household contacts were followed with prevalence surveys for tuberculosis disease for 6 years.

**Results:**

FDA microscopy was positive for a median of 119 (interquartile range [IQR], 47–386) bacteria/µL sputum, which was 5.1% (IQR, 2.4%–11%) the concentration of acid-fast microscopy–positive bacteria (2069 [IQR, 1358–3734] bacteria/μL). Tuberculosis was diagnosed during follow-up in 6.4% (13/209) of contacts. For patients with lower than median concentration of FDA microscopy–positive *M. tuberculosis*, 10% of their contacts developed tuberculosis. This was significantly more than 2.7% of the contacts of patients with higher than median FDA microscopy results (crude hazard ratio [HR], 3.8; *P* = .03). This association maintained statistical significance after adjusting for disease severity, chemoprophylaxis, drug resistance, and social determinants (adjusted HR, 3.9; *P* = .02).

**Conclusions:**

*Mycobacterium tuberculosis* that was FDA microscopy negative was paradoxically associated with greater infectiousness. FDA microscopy–negative bacteria in these pretreatment samples may be a nonstaining, slowly metabolizing phenotype better adapted to airborne transmission.

## Summary

Fluorescein diacetate microscopy was used to assess *Mycobacterium tuberculosis* metabolic activity in pretreatment sputa. Lower results predicted greater infectiousness in transmitting tuberculosis to household contacts. Fluorescein diacetate microscopy–negative bacteria may be better adapted to airborne tuberculosis transmission and consequently more contagious.

(See the editorial commentary by Anthony, on pages 507–8.)

Patients with pulmonary tuberculosis (TB) expectorate sputum that contains heterogeneous subpopulations of *Mycobacterium tuberculosis* (*Mtb*) [1], which has unknown implications for assessing patient infectiousness [2]. Currently, predictions of TB infectiousness are largely based on sputum acid-fast smear microscopy results that are used to guide decisions about infection control measures and to prioritize which patients’ contacts should receive screening, chemoprophylaxis, and surveillance [3, 4]. However, acid-fast microscopy stains *Mtb* bacilli whether they are metabolically active, inactive, or dead [5]. This is important because in vivo and in vitro experiments have suggested that *Mtb* bacteria that have a slowly metabolizing phenotype induced by growth under stress-inducing conditions are most infectious [6–9].

Fluorescein diacetate (FDA) is used in viability assays because it only visualizes cells that produce nonspecific esterase enzymes and have intact cell membranes to retain them, because these enzymes must be present to hydrolyze FDA to its fluorescent form [10]. FDA has been used with sputum microscopy to rapidly and inexpensively predict TB culture positivity [11, 12], and we have found that this can predict the amount of culturable TB in sputum [13] and assess whether early TB treatment is inducing an appropriate treatment response in patients [14, 15].

For the present research, we hypothesized that microscopy with FDA of pretreatment sputum would predict TB infectiousness, as suggested in an editorial [16] commenting on our previous findings. To test this hypothesis, we worked with a group of patients with pulmonary TB who had FDA microscopy performed as previously described [14]. To assess whether FDA microscopy results predicted their risk of transmitting TB, we followed up their contacts and screened those contacts for TB.

## METHODS

### Ethical Considerations

The project had ethics committee approval, all participants gave informed written consent, and all clinically relevant results were provided in writing to participants and their healthcare professionals in collaboration with the local ministry of health, as described elsewhere [14]. Patients received empiric first-line TB therapy with clinic-based direct observation of every dose provided by the national TB program, which follows World Health Organization guidelines [17]. Tuberculosis-related care for patients and their close contacts including diagnostic testing, treatment, and chemoprophylaxis is provided free from direct charges in Peru and was not delayed or modified by study participation.

### Study Participants

Patients were unselected consecutive adults diagnosed with TB that was strongly sputum microscopy-positive (ie, Ziehl-Neelsen grade “+++” or “++,” excluding patients whose samples were “+” or negative [18]). Recruitment took place in shantytowns in Callao, Peru, where the average TB notification rate in these participating clinics during the study period was 194 cases per 100000 people per year. According to local practice, we estimated that the background case notification rate in these communities was 20% higher (ie, 233 cases/100000 people/year) because some cases were treated by other providers [17, 19, 20].

Patients’ contacts were invited to participate if they spent ≥6 hours/week in the patient’s household in the 2 weeks prior to the patient’s diagnosis [21]. Patients and contacts were interviewed to record symptom and demographic data; weight and height were measured; and, for adults, body mass index (BMI) was calculated. Socioeconomic status was assessed by a composite household index in arbitrary units derived by principal components analysis from 13 variables characterizing education, housing conditions, basic services and assets, as described previously [21, 22]. Radiographs were not routinely performed so data were not available [23]. Patient characteristics are shown in Table 1.

**Table 1.** Patient Characteristics Predicting Quantitative Results of Acid-Fast Auramine Microscopy, Fluorescein Diacetate Microscopy, and Culture

Variable	Value	No.^a^	Auramine Microscopy		FDA Microscopy		Quantitative Culture	
			Coefficient	*P* Value	Coefficient	*P* Value	Coefficient	*P* Value
Patient laboratory characteristics								
Auramine microscopy, bacteria/μL, median (IQR)	2069 (1358–3734)	35	NA	NA	**1.3**	**<.001**	**1.1**	**<.001**
FDA microscopy, bacteria/μL, median (IQR)	119 (47–386)	35	**0.49**	**<.001**	NA	NA	**0.66**	**<.001**
Quantitative culture, CFU/μL, median (IQR)	40 (9–80)	33	**0.43**	**<.001**	**0.69**	**<.001**	NA	NA
Time to positive culture, d, median (IQR)	7 (6–10)	34	–0.059	.06	**–0.13**	**.01**	**–0.14**	**.005**
Multidrug-resistant TB, No. (%)	4 (12)	35	–0.015	1	–0.53	.2	–0.47	.3
Patient symptoms								
Productive cough, No. (%)	29 (90)	32	0.13	.7	0.65	.2	0.54	.4
Hemoptysis, No. (%)	17 (53)	32	0.16	.4	0.31	.3	0.18	.6
Cough duration before care seeking, d, median (IQR)	30 (20–60)	23	–0.0058	1	0.04	.9	–0.39	.4
Fever, No. (%)	22 (69)	32	**0.41**	**.04**	0.54	.1	0.47	.2
Night sweats, No. (%)	23 (72)	32	**0.65**	**.001**	**0.88**	**.008**	**0.89**	**.02**
Constitutional symptoms: fevers or night sweats, No. (%)	24 (75)	32	**0.67**	**.001**	**0.95**	**.006**	**1.03**	**.007**
Patient demographics								
Age, y, median (IQR)	26 (22–35)	35	0.0049	.6	0.0014	.9	0.0066	.6
Male sex, No. (%)	20 (57)	35	0.081	.7	0.15	.6	0.46	.1
Body mass index, kg/ m^2^, mean (SD)	21 (2.7)	32	–0.019	.6	0.017	.8	–0.046	.4

Predictors were calculated by univariable linear regression analysis. Values in bold have associations with *P* ≤ .05. Additionally, 2 patients reported previous TB diagnosis and 2 patients did not have a BCG scar, and these variables were not associated with the results of auramine microscopy, FDA microscopy, or quantitative culture (all *P* > .2). Only 1 patient reported having a coexistent chronic respiratory illness, 1 patient reported substance abuse, and no patients had a history of human immunodeficiency virus, diabetes, regular smoking, or heavy alcohol use. All microscopy and quantitative culture data were log-transformed prior to regression analysis (see Methods). Thus, the coefficients indicate the differences in log concentrations (log = base-10 logarithm).

Abbreviations: CFU, colony-forming units; FDA, fluorescein diacetate; IQR, interquartile range; NA, not applicable; SD, standard deviation; TB, tuberculosis.

^a^The column labeled “No.” indicates the number of patients with available data.

### Contact TB Chemoprophylaxis

Isoniazid chemoprophylaxis was provided for 6 months free of charge by the national TB program to contacts aged <15 years, once the national TB program had excluded active TB disease, regardless of any tuberculin skin test (TST) results [23]. Older contacts, especially those aged 15–19 years, were also eligible to receive chemoprophylaxis at the discretion of the national TB program clinician [23].

### Contact Tuberculin Skin Testing

For the current research study, when the patient with newly diagnosed TB was commencing therapy, contacts aged ≥15 years in their household were asked at the time of patient recruitment to undergo screening for asymptomatic latent TB with a TST, as described [24, 25]. However, to encourage participation, our study did not require a TST.

### Contact TB Prevalence Surveys

**Figure 1.**
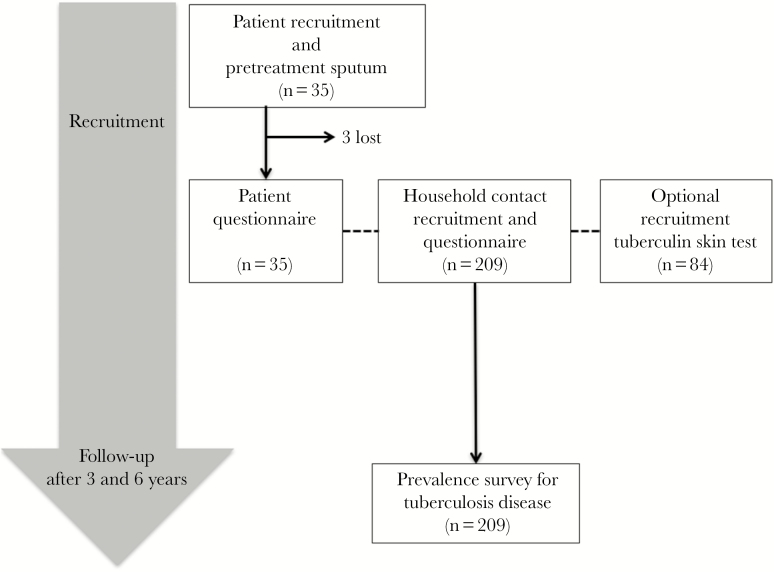
Study flowchart.

At the time of recruitment during 2006–2007, and again during follow-up visits approximately 3 and 6 years later until 2013, participants were asked whether they had TB diagnosed (Figure 1). Self-reported TB episodes were confirmed by checking national TB program records. At these recruitment and follow-up visits, contacts were also asked whether they had symptoms suggestive of TB disease. Contacts with cough, fever, night sweats, or weight loss were requested to provide a sputum sample that we tested with acid-fast auramine microscopy [26] and the microscopic-observation drug-susceptibility (MODS) liquid culture technique [27, 28].

### Sample Processing

Prior to treatment initiation, a pooled single sputum sample was collected overnight for approximately 12 hours at room temperature, and transported at 4°C to our laboratory the following morning. Samples were decontaminated, centrifuged as described previously [14, 26], and immediately smeared onto microscope slides. The pellet suspension was also inoculated for MODS quantitative culture including serial dilutions of each sample, acid-fast auramine microscopy, and FDA microscopy, all performed in triplicate, as described previously [14, 27–30]. All samples were also tested for isoniazid and rifampicin resistance, as described previously [14]. Microscopy techniques together took approximately 1 hour.

### Bacterial Quantification

Visible stained bacteria were counted in 100 consecutive microscopy fields at high power ×1000 magnification with an oil immersion lens. These microscopy counts of bacteria and the numbers of colony-forming units (CFU) per culture well were transformed to the concentration per microliter of sample [14, 29]. All laboratory tests were performed blinded to clinical status and to other test results [14].

### Statistical Analysis

Statistical analysis was done with Stata software version 12, as described previously [14]. Bacterial and CFU counts were exponentially distributed, so were transformed to their base-10 logarithm. Tests were 2-tailed and were performed with a 95% confidence level. Data with normal distributions were summarized by mean and standard deviation (SD) and nonparametric data by median and interquartile range (IQR). Time-to-event Cox regression analysis was used to analyze the hazard ratio (HR) of TB diagnosis in contacts adjusted for household clustering, censored at the time of last follow-up. For the time-to-event Cox regression analysis, secondary TB was a rare outcome and independent variables did not have Gaussian distributions, so all data were analyzed as dichotomous variables above or below the median value for contacts, labeled “high” or “low,” respectively. All univariable analyses, including univariable Cox regression analyses, were performed with nonimputed data. To prevent missing data in >1 variable from reducing the statistical power of multivariable analysis, the primary multivariable analyses were performed with imputed data. Data were imputed by extrapolating results from corresponding laboratory results, or by using the median response from the participants’ questionnaire data. Two multivariable sensitivity analyses were also done: (1) excluding imputed data and (2) censoring follow-up after 5 years.

## RESULTS

### Laboratory Characterization

All 35 pretreatment sputum samples from patients had positive microscopy results for acid-fast auramine and FDA, and had confirmed *Mtb* in culture [14]. Quantitative culture results were obtained for 96% (33/35) of samples because 2 failed due to fungal overgrowth. Quantitative culture CFU results were predicted by both FDA microscopy (coefficient = 0.66; *R*^2^ = 0.46; *P* < .001) and by auramine microscopy (coefficient = 1.1; *R*^2^ = 0.47; *P* < .001; Table 1). Time to positive culture (TTP) in days was significantly associated with quantitative culture results and with FDA microscopy results (both *P* ≤ .01; Table 1). Drug susceptibility testing revealed that 11% (4/35) of patients had multidrug-resistant (MDR) TB. Two patients had isoniazid-monoresistant TB that we classified for statistical analysis as non–MDR-TB.

### FDA Microscopy Results

**Figure 2.**
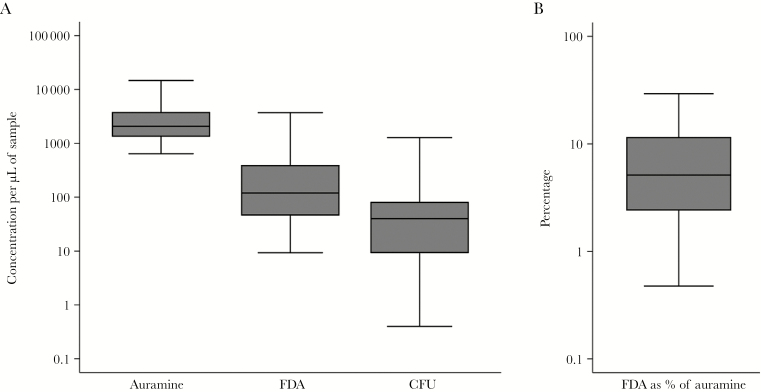
Patients’ pretreatment sputum results. *A*, Distribution of concentrations in sputum of bacilli staining with acid-fast auramine microscopy, bacilli staining with fluorescein diacetate (FDA) microscopy, and colony-forming units (CFU) in quantitative culture. *B*, Distribution of FDA microscopy–positive bacterial concentration as a percentage of auramine microscopy–positive bacteria per microliter.

The median concentration of FDA microscopy–positive bacteria/μL was 119 (IQR, 47–386; Figure 2). This median FDA microscopy–positive bacterial concentration was 5.1% (IQR, 2.4%–11%) of the concentration of auramine microscopy–positive bacteria/μL (2069 [IQR, 1358–3734]; Figure 2). The median concentration of CFU/µL was 40 (IQR, 9–80; Figure 2). This CFU concentration was a median 1.8% (IQR, 0.42%–2.2%) of the concentration per microliter of auramine-staining bacteria. The between-patient variability of concentrations of FDA-stained bacteria and CFU were both more than double the between-patient variability of concentrations of auramine-positive microscopy (IQR of the log-concentrations 0.92 and 0.93 vs 0.44, respectively; Figure 2).

### Patient Characteristics

The patient characteristics and their associations with the results of sputum laboratory testing are shown in Table 1. Constitutional symptoms (night sweats and/or fevers) were associated with high results of: auramine microscopy, FDA microscopy, and culture CFU (all *P* ≤ .04; Table 1). No patients had evidence of extrapulmonary or disseminated TB.

### TB Infection in Contacts

At the time of patient recruitment, 209 of the patients’ contacts were also recruited (Figure 1). Contact characteristics are summarized in Table 2. These contacts had a median age of 20 (IQR, 10–32) years, 50% (105/209) were male, and the mean BMI was 25 (SD, 5.2) kg/m^2^. Only 40% (84/209) of the contacts underwent a TST. This was because 38% (80/209) of the contacts were ineligible because they were aged <15 years and 35% (45/129) of the remaining contacts declined. Seventy percent (59/84) of TST results were positive. Positivity was not associated with any laboratory or clinical characteristics (data not shown), but confidence intervals (CIs) were wide because so few participants had a TST.

**Table 2.** Characteristics Predicting Secondary Tuberculosis Disease in Contacts

Characteristic	Patients, No.^a^	Contacts, No.^a^	Hazard Ratio	(95% CI)	*P* Value
Patient sputum laboratory characteristics					
Auramine microscopy above median (vs below median)	35	209	0.75	(.21–2.6)	.6
FDA microscopy above median (vs below median)	35	209	**0.26**	(**.08–.90**)	**.03**
Quantitative culture above median (vs below median)	33	201	2.4	(.74–7.5)	.2
MDR (vs non-MDR)	35	209	2.6	(.39–17)	.3
Patient disease characteristics					
Productive cough in patient (vs no productive cough)	32	203	**High**	**High**	**<.001**
Hemoptysis in patient (vs no hemoptysis)	32	203	3.9	(.85–18)	.08
Fever in patient (vs no fever)	32	203	4.7	(.81–27)	.09
Cough duration before care seeking above median (vs below median)	23	133	0.44	(.10–2.0)	.3
Household characteristics					
Socioeconomic score poorer than median (vs less poor)	35	209	**3.8**	(**1.1–13**)	**.03**
No. of contacts/household above median 5 (vs less than median)	35	209	1.0	(.27–4.0)	.1
Contact characteristics					
Household contact completed chemoprophylaxis (vs not completed)	…	209	**Low**	**Low**	**<.001**
Male sex (vs female)	…	209	0.61	(.21–1.8)	.4
Age ≤15 y (vs >15 y)	…	209	0.29	(.10–1.3)	.1
Age above median 20 years (vs below median)	…	209	1.8	(.71–4.5)	.2
BMI above median 25 kg/m^2^ (vs below median) for adults	…	96	1.3	(.34–5.1)	.7

All the variables in Table 1 and the contact characteristics were tested for associations predicting secondary TB disease in contacts by univariable Cox regression analysis. Additionally, no household contact had a history of human immunodeficiency virus or diabetes. Imputation of missing data was only performed for multivariable analyses, so the analyses in this table only used original, nonimputed data (see Methods). See Table 3 for multivariable regression analysis. Values in bold have associations with *P* ≤ .05. The contact characteristics are also reported here stratified for contacts of patients with high vs low FDA microscopy results: completed chemoprophylaxis, 11/110 (11%) vs 8/99 (8%); male sex, 54/110 (49%) vs 51/99 (52%); age >15 years, 42/110 (38%) vs 38/99 (38%); age greater than median years, 54/110 (49%) vs 44/99 (44%); BMI greater than median, 25/50 (50%) vs 23/46 (50%).

Abbreviations: CI, confidence interval; FDA, fluorescein diacetate; MDR, multidrug resistant; TB, tuberculosis.

^a^The columns labeled “No.” indicate the number of patients with available data.

### TB Disease in Contacts—Univariable Analysis

**Figure 3.**
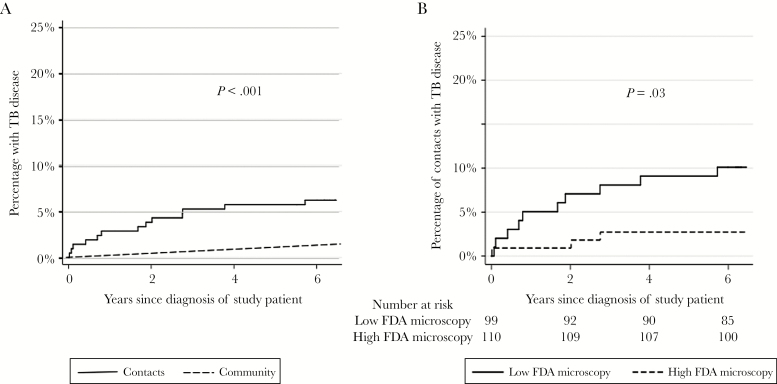
Kaplan-Meier time-to-event curves for patients’ household contacts being diagnosed with tuberculosis (TB) disease. A, Compared with the background community rate of 233 new cases of TB per 100 000 people per year. B, Analyzed according to whether the patients’ pretreatment sputum fluorescein diacetate (FDA) microscopy result was above (high) or below (low) the median. P values indicate the results of Cox regression analysis (see Results).

TB disease was diagnosed in 6.2% (13/209) of contacts during follow-up that lasted a median of 6.3 (IQR, 6.2–6.5) years. This was significantly higher than the background community rate (HR, 4.0 [95% CI, 1.9–8.4]; *P* < .001, Figure 3A). All the variables shown in Table 1 and the contact characteristics were tested for associations with secondary TB disease in contacts using univariable Cox regression analysis. FDA microscopy–positive concentrations of bacteria per microliter were analyzed around the median value as high (median, 386 [IQR, 163–601]) or low (median, 22 [IQR, 9–47]). All variables with biologically plausible or statistical evidence (*P* < .1) of associations with secondary TB in contacts are shown in Table 2. Contacts of patients with low FDA microscopy results were 4-times more likely to have TB disease (unadjusted HR, 3.8 [95% CI, 1.1–13]; *P* = .03; Table 3 and Figure 3B). Specifically, 2.7% (95% CI, .57%–7.8% [3/110]) of contacts exposed to patients with high FDA microscopy results developed TB disease during follow-up, which did not significantly differ from the background community rate (*P* = .4; Figure 3). In contrast, 10% (95% CI, 5.0%–18% [10/99]) of contacts exposed to patients with the low FDA microscopy results developed TB disease during follow-up (Figure 3B).

**Table 3.** Patient Fluorescein Diacetate Microscopy Predicting Secondary Tuberculosis Disease in Contacts

Variable	Primary Analysis Including Imputed Data (n = 35 Patients; n = 209 Contacts)			Sensitivity Analysis 1 Excluding Imputed Data (n = 32–35 Patients; n = 201–209 Contacts)			Sensitivity Analysis 2 Censored at 5 Years (n = 35 Patients; n = 209 Contacts)		
	HR	(95% CI)	*P* Value	HR	(95% CI)	*P* Value	HR	(95% CI)	*P* Value
FDA low (vs high), unadjusted analysis	3.8	(1.1–13)	.03	3.8	(1.1–13)	.03	3.4	(.96–12)	.059
FDA low (vs high) adjusted for:									
Higher than median quantitative culture	8.1	(3.1–21)	<.001	8.2	(3.1–21)	<.001	7.8	(3.0–20)	<.001
Productive cough in patient	4.5	(1.4–15)	.01	3.9	(1.1–14)	.03	4.1	(1.2–14)	.03
Poorer than median socioeconomic score	4.2	(1.4–13)	.01	4.2	(1.4–13)	.01	3.9	(1.2–12)	.02
Chemoprophylaxis completed	3.8	(1.0–14)	.04	3.8	(1.0–14)	.04	3.3	(.9–13)	.07
Quantitative culture, productive cough, socioeconomic score, and chemoprophylaxis	5.5	(1.8–17)	.003	5.3	(1.8–16)	.003	4.6	(1.6–13)	.005
Quantitative culture, productive cough, and chemoprophylaxis	7.1	(2.5–20)	<.001	6.7	(2.4–18)	<.001	6.8	(2.4–19)	<.001

Results were calculated by multivariable Cox regression analysis adjusting the association of FDA microscopy with the variables that in univariable analysis significantly predicted secondary tuberculosis (TB) disease in contacts (shown in Table 2). High vs low auramine microscopy results were not associated in univariable analysis with secondary TB (*P* = .6) and, if despite this auramine microscopy results were included in the adjusted analyses, then the pattern of significance was unchanged. The presence vs absence of multidrug-resistant (MDR) TB was not associated in univariable analysis with secondary TB (*P* = .3) and, if despite this MDR-TB was included in the adjusted analyses, then the pattern of significance was unchanged. The primary multivariable analysis included 5 imputed data for quantitative culture and productive cough (see Results section). Sensitivity analysis 1 excluded the imputed data. Sensitivity analysis 2 censored all follow-up at 5 years, causing 1 late case of secondary TB to be excluded.

Abbreviations: CI, confidence interval; FDA, fluorescein diacetate; HR, hazard ratio.

**Figure 4.**
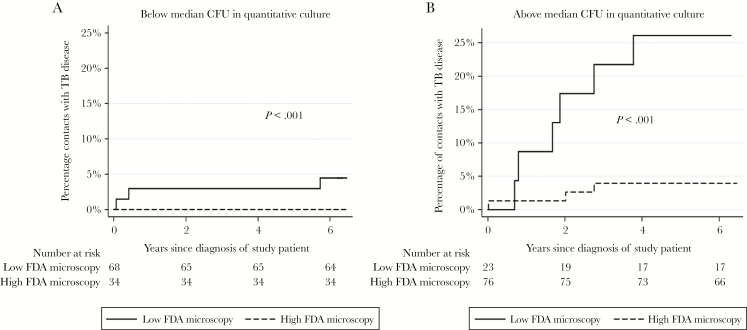
Kaplan-Meier time-to-event curves for patients’ household contacts being diagnosed with tuberculosis (TB) disease analyzed according to whether the study patients’ pretreatment sputum fluorescein diacetate (FDA) microscopy result was above (high) or below (low) the median concentration. *A*, Patients with quantitative culture results (in colony-forming units [CFU]) below the median concentration. *B*, Patients with quantitative culture results above the median concentration. *P* values indicate the results of Cox regression analysis.

TB disease was more likely in contacts of patients who reported having a productive cough and in households with poorer than median socioeconomic score (both *P* ≤ .03; Table 2). Chemoprophylaxis was taken by 9.1% (19/209) of contacts, all aged <15 years, none of whom developed TB disease. Constitutional symptoms (fever and/or night sweats) in patients was associated with FDA microscopy results, and consequent impact on diagnostic delay may influence TB transmission [31, 32], but including either or both variables in all multivariable regression analyses did not affect the significant association between lower than median FDA microscopy results and increased secondary TB. Tuberculosis disease in contacts was not associated with high vs low auramine microscopy results (*P* = .7; Table 2), but all patient samples were strongly auramine microscopy positive. Although in univariate analysis, quantitative culture CFU did not predict TB disease (*P* = .2; Table 2), the association of FDA microscopy and secondary TB disease in contacts was modified by the patient’s corresponding quantitative culture CFU results (*P* < .001; Figure 4). Specifically, contacts of patients with high CFU concentrations and low FDA microscopy results had the highest risk of developing TB disease (unadjusted HR, 7.4 [95% CI, 2.7–20]; *P* < .0001; Figure 4).

### TB Disease in Contacts—Adjusted Multivariable Regression Analyses

For the primary multivariable Cox regression analysis (Table 3), 5 missing data points were imputed. Two patients had missing CFU data because of fungal overgrowth, and high/low CFU categorization was imputed from days to culture positivity and auramine microscopy results. Three patients did not report whether they had productive cough, and this was imputed as the median response.

The association between low FDA microscopy results predicting increased risk of TB disease in patients’ contacts maintained statistical significance after adjusting for quantitative culture CFU results (HR, 8.1 [95% CI, 3.1–21]; *P* < .001), disease severity indicated by productive cough (HR, 4.5 [95% CI, 1.4–15]; *P* = .01), socioeconomic score (HR, 4.2 [95% CI, 1.4–13]; *P* = .01), and chemoprophylaxis (HR, 3.8 [95% CI, 1.0–14]; *P* = .04), and also after adjusting for all these variables (HR, 5.5 [95% CI, 1.8–17]; *P* = .03) (Table 3).

Sensitivity analyses that excluded all imputed data or had follow-up censored after 5 years (that excluded the last episode of secondary TB in contacts that occurred 5.7 years after index case diagnosis and could have been unrelated to that exposure) had almost identical results to the analyses above (Table 3).

Post hoc exploratory analysis of TTP data considered patient cultures as faster or slower than the median value of 7 days. Faster TTP predicted a significantly increased risk of secondary TB in contacts (HR, 4.5 [95% CI, 1.1–19]; *P* = .04). Lower than median FDA microscopy–positive concentrations predicted an increased risk of secondary TB disease in contacts independent of TTP (HR, 3.9 [95% CI, 1.1–13]; *P* = .03).

## DISCUSSION

In strongly microscopy-positive sputum samples collected before TB treatment, the proportions of FDA-stained and culturable *Mtb* bacteria varied considerably between patients. These findings are clinically significant because patients with low FDA microscopy results were most infectious, as demonstrated by the finding that their household contacts were considerably more likely to develop TB disease, including when adjusted for confounding variables and in sensitivity analyses.

Sputum from patients with pulmonary TB is known to contain heterogeneous subpopulations of *Mtb* bacteria [1, 33, 34]. Culture techniques used for diagnosing TB do not generally identify these individual subpopulations because results are determined by the overall quantity of bacteria as well as the fastest-growing subpopulation. A subpopulation of particular interest is persister cells, a term used in the context of chemotherapy to describe *Mtb* that persists in sputum in a slowly metabolizing, nonreplicating state [35]. In pretreatment sputum, most *Mtb* bacteria are believed to be in a persister-like state, as they cannot be cultured using conventional techniques and contain lipid bodies, both features of the phenotypic variant of *Mtb* bacteria that are slow growing or nonreplicating [1, 33, 36]. Furthermore, *Mtb* in persister-like states has been demonstrated not to fluoresce with FDA, unless resuscitation-promoting factors are added [37]. Nonfluorescence with FDA could be explained by loss of cell membrane integrity, changes in the cell wall effecting FDA penetration, or low esterase activity when in a slow metabolizing state [35, 38].

We found that patients were approximately 4-times more likely to cause TB disease in their household contacts if they had low concentrations of FDA microscopy–positive *Mtb* in their sputum. *Mtb* persister-like states develop in response to stressful environments such as nutrient deficiency or hypoxia [39], and persister-like phenotypes of *Mtb* are thought to be better adapted to surviving harsh environments such as during airborne transmission [1]. This increased infectiousness of *Mtb* in persister-like states has been demonstrated by growing *Mtb* under hypoxic or nutrient stress. Compared with *Mtb* grown under conventional aerobic conditions, in vivo stress-induced *Mtb* caused 10 times greater airborne infectiousness to guinea pigs and an altered immune response that increased pathogenicity to mice, and in vitro this increased invasion of epithelial cells and alveolar macrophages [6–9]. We hypothesize that in our study, which only included strongly sputum microscopy–positive TB, patients with low concentrations of FDA microscopy–positive *Mtb* in their sputum had high concentrations of persister-like mycobacteria that were better adapted to airborne transmission, so were more infectious. This hypothesis warrants specific research, in addition to confirmation of the association with infectiousness in larger studies and with other patient groups.

We found considerable variability in the proportion of FDA-stained *Mtb* between different patients’ pretreatment sputa. Most *Mtb* in sputum did not fluoresce with FDA microscopy, and could indicate dead bacteria. However, the most infectious patients in this study were those with low numbers of FDA-staining bacilli but high colony counts in quantitative culture. This indicates that a large proportion of FDA microscopy–negative *Mtb* were not dead but rather had a phenotype associated with greater infectiousness. It is possible that the use of resuscitation factors [37, 40] or more prolonged culture [33] may cause higher concentrations of *Mtb* to be identified in both FDA microscopy and quantitative culture. Processing sputum without decontamination may also have this effect, but would increase fungal overgrowth of cultures, which confounds CFU quantification [29]. As expected, patients with more constitutional symptoms had significantly higher sputum *Mtb* concentrations, probably due to having more extensive pulmonary TB disease [41].

We found that the risk of secondary TB disease was higher for contacts of patients with high quantitative culture results, whether indicated by high CFU (in adjusted analysis) or rapid culture TTP (in univariable analysis), both indicators of mycobacterial load. Generally, smear positivity is also associated with greater infectiousness, but a meta-analysis of risk factors for TB transmission required thousands of participants to demonstrate that patients with concentrations of *Mtb* in their sputum sufficient for positive acid-fast microscopy had a 1.4 times (95% CI, 1.2–1.6) increased risk of causing TB disease in their contacts compared with acid-fast microscopy–negative patients [42]. This finding does not contradict the results of our study, which included only strongly microscopy-positive patients. In contrast to the 1.4 times greater TB infectiousness in acid-fast microscopy-positive vs acid-fast microscopy-negative patients in this meta-analysis, our results suggest that FDA microscopy results had a considerably larger effect that was independent of the results of microscopy, CFU, TTP, or other potentially confounding variables. In fact, our study demonstrates that contacts of patients who were acid-fast microscopy positive, but had high concentrations of fluorescing bacteria in FDA microscopy, had no higher risk than the background rate in their community.

Contacts of patients with pulmonary TB disease are at high risk of developing TB disease and are often provided with chemoprophylaxis. This policy is consistent with the apparent protective effect of chemoprophylaxis in our study. However, in most high-TB-prevalence settings, resources are insufficient for comprehensive contact tracing within and outside the home [43]. Provision of chemoprophylaxis to all household contacts may be appropriate in some settings. However, a low-cost tool, such as FDA microscopy, may identify the most infectious individuals and allow better prioritized contact tracing for TB disease screening and prevention than the current use of conventional sputum acid-fast microscopy results.

In addition to studying infectiousness to cause TB disease in contacts, we also aimed to assess infectiousness to cause asymptomatic latent TB in contacts. However, most of the contacts in our study were too young to be eligible for, or declined, a TST. This low uptake of TSTs reduced the statistical power so much that we were unable to meaningfully test for associations with asymptomatic latent TB. Furthermore, concurrently we discovered that the majority of healthy adults in these communities were already TST positive, even without living with a known TB patient [22, 44]. Thus, living with a patient newly diagnosed with pulmonary TB increased the risk of TST positivity by <50% [22, 44]. Therefore, to adequately test the hypothesis that FDA-negative *Mtb* in sputum also predicts infectiousness for causing asymptomatic latent TB would require much larger future studies, ideally using the more specific interferon-γ release assays in a setting with a low background rate of TB infection [44–46].

The main limitation of this research is that FDA microscopy was only assessed in *Mtb* strongly microscopy-positive samples. Therefore, in future research, FDA microscopy should be assessed in samples with weakly positive and negative acid-fast microscopy results, which may be facilitated by concentrating *Mtb* in sputum by filtration [47] or centrifugation [29]. To reduce the risk of false-positive FDA microscopy results due to other viable bacteria within the sample, we decontaminated all sputum samples, which is known to kill some *Mtb* [48]. Whether decontamination selectively affected the viable subpopulation that was not stained by FDA is unknown and warrants future research. However, this would be expected to affect all samples equally because the same protocol was followed for all the specimens processed, and should also similarly affect the quantitative culture results. Therefore, decontamination could not explain the contrasting results demonstrated between FDA microscopy and quantitative culture predicting infectiousness. Another limitation is that the *Mtb* TB strains were not available to use molecular fingerprinting to confirm whether any of the secondary TB cases in contacts were caused by TB patients other than the index case recruited to our study [49, 50]. However, our conclusions were supported by a sensitivity analysis excluding the last episode of secondary TB in case that late case was unrelated to the index patient (and another sensitivity analysis excluding 5 imputed data points).

In conclusion, the inexpensive, rapid, low-technology technique of FDA microscopy revealed that only a small and variable subpopulation of *Mtb* in sputum was FDA microscopy positive. This study provides evidence that among strongly sputum microscopy–positive patients, those with low concentrations of FDA microscopy–positive *Mtb* were most infectious to cause TB disease in their contacts. This supports the hypothesis that a subpopulation of *Mtb* that do not stain with FDA, such as persister-like bacteria, are most infectious to transmit TB disease. Thus, FDA microscopy identified a high-risk group of TB patients whose contacts particularly need interventions to prevent and screen for secondary TB disease.

## Notes

***Author contributions.*** All authors participated sufficiently in the work, the data analysis, and/or the preparation of the manuscript, and all have reviewed and approved the manuscript as submitted to take public responsibility for it. Conception, hypotheses delineation, and study design: J. M. S., T. V., R. M., R. H. G., C. A. E. Data acquisition, analysis, or interpretation: S. D., J. M. S., M. A. B., M. A. T., T. V., R. M., W. Q., N. D., E. S. R., R. H. G., C. A. E. Writing the article or substantial involvement in its revision: S. D., R. H. G., C. A. E. The corresponding author had full access to all study data and was responsible for the decision to publish.

***Acknowledgments.*** The authors are grateful for the contributions of the research team, not all of whom meet the criteria to be coauthors; for expert administrative support from Silvia Carrera and Maribel Rivero; and to the patients, who agreed to put aside TB-associated stigma to participate in this project. The authors are particularly grateful to the local and national Peruvian Ministry of Health for collaboration and support.

***Disclaimer.*** The study funders had no role in study design, data interpretation, or writing of this report.

***Financial support.*** This work was supported principally by the Wellcome Trust Fellowship 105788/Z/14/Z. Specific activities and researchers were also supported by the Wellcome Trust (award numbers 057434/Z/99/Z, 070005/Z/02/Z, and 078340/Z/05/Z); the Joint Global Health Trials Consortium of the Medical Research Council, UK; UK-AID and the Wellcome Trust (award number MR/K007467/1); the Bill & Melinda Gates Foundation (award number OPP1118545); the Imperial College Biomedical Research Centre; the World Health Organization; and the charity Innovation for Health and Development (IFHAD).

***Potential conflicts of interest.*** All authors: No potential conflicts of interest. All authors have submitted the ICMJE Form for Disclosure of Potential Conflicts of Interest. Conflicts that the editors consider relevant to the content of the manuscript have been disclosed.
